# Access to HIV Care and Resilience in a Long-Term Conflict Setting: A Qualitative Assessment of the Experiences of Living with Diagnosed HIV in Mogadishu, Somali

**DOI:** 10.3390/ijerph14070721

**Published:** 2017-07-05

**Authors:** Asli Kulane, John O. A. Owuor, Douglas Sematimba, Sacdia Abdisamad Abdulahi, Hamdi Moalim Yusuf, Lul M. Mohamed

**Affiliations:** 1 Equity and Health Policy Research Group, Department of Public Health Sciences, Karolinska Institute, Tomtebodavägen 18a, Widerströmska Huset, 171 77 Stockholm, Sweden; douglas.sematimba@ki.se; 2 Centre for Global Health (CGH), Trinity College Dublin, The University of Dublin, College Green, Dublin 2, Ireland; Owuorj@tcd.ie; 3 Ministry of Health, Mogadishu, Somalia; sacdiaas@hotmail.com; 4 Banadir Hospital, Women and Child Care Section, Mogadishu, Somalia; drhamdimy@gmail.com (H.M.Y.); drlul@hotmail.com (L.M.M.)

**Keywords:** Somali, conflict, health system, resilience, fragile, access, government, NGO

## Abstract

Background: Human Immunodeficiency Virus (HIV) continues to take a heavy toll on the lives of many people, with the worst impact on health and wellbeing for the affected individuals in fragile states. The HIV situation in Somalia is not clearly known and experiences of the people living with HIV in this war-torn region are often unexpressed. This pilot qualitative study sought to explore the experiences of people diagnosed with HIV living in Mogadishu, and their resilience in access to care and social support. Methods: Participants were recruited through drug dispensers at the HIV clinic in Banadir Hospital. Face-to-face in-depth interviews were conducted in Somali in May 2013 among patients who were receiving antiretroviral therapy (ART) from the HIV clinic in Mogadishu. These were tape-recorded, transcribed, and translated for content analysis. Results: Three women and four men who were living with HIV shared the following narratives. Their perception was that they had either got HIV from their spouses or through health care contamination. They were very knowledgeable about the realities of HIV, how the medication works, nutritional requirements, and drug adherence. They were always willing to go an extra mile to secure a good life for themselves. However, the external HIV stigma impacted their access to care. They faced challenges in their homes and at work which compelled them to seek support from non-governmental organizations (NGOs) or close family members. This stigma often affected their disclosure to the wider community due to the uncertainty of the repercussions, leading to a life of extreme loneliness and financial difficulties. The participants’ coping mechanisms included living together and starting their own NGO for support with very strong optimism about their prognosis. Conclusions: The people diagnosed with HIV living in Mogadishu are highly knowledgeable about HIV transmission, the realities of living with a diagnosed HIV infection, and the efficacy of HIV treatment. Our small sample suggests adequate access to ART through NGOs. However, widespread HIV stigma limits HIV status disclosure to families and communities, which creates a risk of self-isolation and ill health. Still, affected individuals have developed resilient mechanisms for managing the risks. They strive to remain employed for economic security, adhere to HIV treatment, engage in support groups, and maintain the utmost optimism about their prognosis.

## 1. Background

Human Immunodeficiency virus (HIV) continues to cause devastating effects including stigma and isolation to individuals and communities nearly four decades since the disease was first isolated. An estimated 36.9 million people globally were living with HIV in 2014, the vast majority of whom resided in low- and middle-income countries (LMICs). An estimated 34 million people have died from acquired immune deficiency syndrome (AIDS)-related causes so far, including 1.2 million in 2014 [1]. 

Since its discovery, varying views and opinions have been expressed in different communities concerning HIV. Even though some communities have accepted the science of HIV transmission, treatment, and prevention, many still live in denial with varying levels of stigma, myths, and misconceptions that have caused constant depression among those affected [2,3]. The World Health Organization (WHO) has recommended full access to Antiretroviral therapy (ART) for all in need, but reaching all eligible people with treatment remains a huge global challenge. By mid-2015, 15.8 million people living with HIV were receiving ART globally and, of these, close to 13.5 million live in LMICs. By the year 2030, 21 million AIDS-related deaths and 28 million infections will have been averted expanding ART and prevention choices to people living with HIV [1].

There is limited knowledge about the HIV pandemic in Somalia, especially in Mogadishu, where prevailing insecurity limits any data collection efforts. The only data on HIV that was availed in this region was in 2004, which stated that the prevalence of HIV was 0.7% to 1%. This makes it difficult to make proper estimates, because the analysis of data from Voluntary Counselling and Testing Centers in addition to Tuberculosis (TB) clinics showed that 18% of TB patients and 5.2% of female sex workers were infected with HIV [4]. These isolated groups that have been targeted make it difficult to understand the real situation of HIV; yet, the conditions that drive the spread of the HIV epidemic are observed to be existent, i.e., increased trade, migration, and the sprouting of sex work at borders and ports [4].

For other nations that have been relatively peaceful, circumstances surrounding access to HIV care and experiences of living with HIV have been studied [5,6]. Similar studies in Somalia were not conducted because of the civil strife and unrest that has brought continuous fragility in the country, particularly in the Mogadishu region. Despite the drafting of the national HIV strategy, Somalia still faces challenges of low coverage of HIV services with a low capacity for monitoring and evaluation, and a poor supply of food and medicines in addition to the country’s insecurity [7]. With the consistent realities of poverty come several challenges in this most eastern nation in the horn of Africa that has been ravaged by war for several years [8,9,10,11]. This pilot qualitative study sought to explore the experiences of people living with diagnosed HIV in Mogadishu and their resilience in access to care and social support.

## 2. Methods 

### 2.1. Study Design and Data Collection

This study was conducted in Mogadishu in May 2013. Participants were recruited through the ART dispensers at an HIV clinic as they came for refills of their medication. The study was approved by the hospital since there is no active ethical review board. This allowed the study to proceed, provided the participants gave informed consent. Participants were asked whether they were interested in taking part in the study and those who showed interest were given detailed information on the study, described the principles of voluntary participation, anonymity and the right to withdraw without any consequences for the participant. Informed consent was obtained orally from those who agreed to participate and the consent proceedings were audio recorded. One of the participants declined to be audio recorded due to confidentiality concerns and their interview was handwritten.

All interviews took place in a medicines storage room at the local hospital, except for one female participant whose interview was conducted in an office within the hospital. The in-depth interviews (IDIs) were conducted in Somali by the first author.

### 2.2. Data Analysis 

The data from the audio tapes were transcribed and translated from Somali to English. The data from the handwritten interview was also translated into English. The researchers read and familiarized themselves with the data from the interviews and the interpretation was done using content analysis as described by Graneheim and Lundman by the first and third author [12]. The findings were shared with the rest of the team in Mogadishu for validation. The researchers developed codes for the meaning units that were put into categories from which the themes described in the next section emerged.

## 3. Results

### 3.1. Study Participants 

It was extremely challenging to access participants for our study due to the insecure situation and HIV stigma in Mogadishu. Face-to-face interviews require some sense of common trust between the participants and the researcher. However, it is difficult to establish effective trust under the prevailing circumstances in Mogadishu in a short period of time. Despite these challenges, we managed to conduct in-depth, face-to-face interviews with seven individuals living with HIV, including three women and four men. All the participants were on highly active antiretroviral treatment (HAART) and were accessing their HIV medication supplies from a treatment center in Mogadishu. Prior to the establishment of the local facility, the participants said they had to travel to another city called Merca for their medication. The participants had lived with diagnosed HIV for varied timeframes ranging from one year to seven years prior to the study. All the participants had experienced death of loved ones from HIV-related conditions. They had lost a wife or a husband, and/or children to HIV. At the time of the study, the participants said they were living in what we call a community of positives, a ‘community of people with HIV’. Thus, they lived in close proximity to each other for peer social support. Some of them shared accommodation and were able to support each other even more. 

Key themes emerged from the participants’ narratives regarding what it meant for them to live with HIV in a complex and challenging social, political, and economic context, as discussed further below. 

### 3.2. Perceptions about HIV 

When asked about how they believed they got infected by HIV, our study informants believed that they acquired HIV through two main ways; through their previous spouses, most of whom had died of the disease, or through contamination through health care. However, the participants emphasized that they could not tell with certainty how they got the disease. The female participants, for example, said that they got infected when giving birth or when seeking other medical care that involved the risk of bodily fluid transfer such as dental care. In addition, there were reports of occupational exposure as illustrated below.

Interviewee: I suspect … she was part of those working in the rescue services, picking up injured people when Mogadishu was shelled during the war … there must have been blood exchange. Then there was a time she had dental procedure as well. Maybe she got infected then. She also got some of our babies through traditional birth attendance, where they used non-sterile equipment. I am not sure where HIV followed us from. We only found out in testing …

The above extract from a male participant describing how he believed his wife got infected illustrates uncertainty about the source of HIV infection, and how the participants concerned acquired the disease. It provides an account of perceived potential avenues of infection, which also reflects awareness of the risks of infection at the time of the study. The above extract also makes reference to HIV being a disease that followed the relevant couple. In other words, they did not go out of their way to get HIV. They kept their distance but still HIV ‘followed’ them. The extract thus suggests a sense of inevitability, that HIV was an eventuality that occurred despite the individual’s efforts. Additionally, the participants noted that HIV caused the degradation of the social fabric of the society. For instance, there were reports of families rejecting their own members who had HIV, including young children. On the other hand, caring for family members with HIV also depleted material and emotional resources of affected families, leading to poverty and hopelessness. In this way, HIV broke down the social networks from different angles. 

### 3.3. Treatment Experiences

The participants noted that they all had access to treatment, and that it was free of charge. Furthermore, HIV treatment was available locally in Mogadishu at the time of the study, despite persistent insecurity. As highlighted above, this was a great improvement in the lives of people living with diagnosed HIV in Mogadishu. Previously, some of the participants said they had to travel to another city, Merca, to obtain treatment. Although HIV treatment was a recent intervention in Mogadishu, the participants further noted that HIV testing had been available in Mogadishu for a long time, mainly provided by non-governmental organizations. In fact, the entire HIV testing and treatment process was or had been provided by non-governmental organizations (NGOs). 

When asked about their views about adherence to their HIV medication, the participants said that they were adhering to the treatment as advised by their physicians. All of them reiterated awareness that they would suffer serious health setbacks if they did not adhere to their treatment as illustrated here, *Interviewee: I take it on time. May be 10 min delay if I am over carried* (implying distracted)*.* However, the participants also reported knowledge of their peers in the HIV support groups who did not conform to the physicians’ recommendations on treatment adherence, highlighting the challenges of treatment adherence. 

Conversely, the participants were aware that HIV treatment was not a cure. They said that the treatment merely relieved their illness and gave them the opportunity to go on with their lives. Calling HIV treatment *Kalmanti* (calming the situation), the participants believed that the medications only minimized the potential impact of HIV but they still had to live with HIV for as long as they lived, despite strictly adhering to medication. However, this observation should not be misunderstood for a lack of appreciation of the effectiveness of highly active antiretroviral treatment (HAART). In fact, the participants emphasized that that they were glad to be alive because they had access to effective treatment. The above observations thus suggest that the participants accepted the reality that there was still no cure for the disease. 

We also found that a common view that the medications were also toxic, suggesting a high level of awareness about HIV treatment side effects and requirements among the people we interviewed. For example, a participant noted that the drugs were also damaging their bodies, as illustrated below.

Interviewee: They (medication) have their own weight. For example, they need one to eat well, nutritious food. But we don’t have that, so the medicine is wearing us out. Without the right food, the medicine makes one weak. It protects you yes from that disease, but it also weakens the rest of the body.

The above excerpt indicates that the participant was aware of the prerequisites for successful treatment. The extract illustrates the awareness of the need for good nutrition for people on HIV treatment, suggesting that most of the participant’s peers could not afford balanced diets. This implies that other factors were contributing to the fact the participants could not afford nutritious food, hence they took their medications in full awareness that they risked significant side effects due to their socioeconomic circumstances that prevented them from consuming the healthy diets that they needed to survive well on medication. 

### 3.4. HIV Stigma

The narratives of our participants highlighted widespread HIV stigma in Mogadishu. All of the participants said that they had been stigmatized at some point in their life with diagnosed HIV. For instance, all three female participants in our study had been chased away from their homes when their relatives discovered that they had HIV, as illustrated below.

*Interviewee: When I found out I had HIV, the family divided into two and some did not want me around. I then came to my relatives in the south, there they have farms and I could get nutritious food. I came from the middle of the country, mainly dry. But the relatives in the south stigmatized me. When they found out I had HIV, they burnt up my belongings. They gave me 50,000 shillings and told me to go and ‘defend myself in Mogadishu’*.

(meaning to go and fend for herself in Mogadishu)

The above extract also emphasizes another key finding, that HIV caused internal relocation/migration from participants’ original areas of residence to Mogadishu. People living with HIV seemed to move to where they felt it was safe to live with HIV, mainly in close proximity to their peers in Mogadishu. Thus, HIV caused internal relocation to perceived ‘safer’ places. They felt safe in Mogadishu by relying on the fact that people in the new neighborhood did not know about the infected person’s HIV status. Some of the participants said that some landlords, for example, were not willing to rent houses to people with HIV because other tenants would run away. Consequently, a person living with diagnosed HIV was compelled to pay a higher rent to cover any losses in income that a landlord willing to accommodate them would incur as a result of other tenants deserting the building due to HIV stigma.

Interviewee: Now my rent is 150 US dollars per month. Very expensive! … I live in a five-room house. Other tenants in the building left when they discovered I am HIV positive. But my landlord said, you either pay the rent for the whole building or you move out. So we agreed that I pay up for the whole block. I decided to cover the 100 dollars, rent all the rooms. We [people with diagnosed HIV] have nowhere to go. Since the landlady was so nice [willing to accommodate him], I decided to remain, at least she gave me an option to stay. Others chase you away. So now I have five rooms to receive others [living with diagnosed HIV].

Our finding, as illustrated by the above extract, suggests that life with HIV in Mogadishu is an isolated one, accompanied with extreme financial difficulties. The requirement to pay a higher than normal rent for accommodation for people living with HIV, most of whom are poor and unable to afford even a decent meal as alluded to above, suggests that HIV is causing further destitution among those knowingly living with it. Furthermore, as some of our participants noted, being diagnosed as HIV positive also led to the loss of jobs or sources of income.

Interviewee: I worked in a market. Used to sell clothes. But I stopped when they found I had HIV. People here are less informed about HIV. So there is a lot of stigma. No one is willing to buy “HIV man” clothes. I therefore stopped working. There is a lot of misconceptions about HIV. People think one can get it through food, clothes, and things like that.

The above extract illustrates the socioeconomic impact of HIV stigma on those diagnosed with HIV in Mogadishu. It also highlights possible and widespread misconceptions about HIV by the wider community in Mogadishu. Similar to other social contexts, HIV stigma in Mogadishu has led to the concealment or selective disclosure of HIV positive status by those knowingly living with the disease to their family members or friends. Although disclosure is key in accessing social support, our participants observed that it was better to conceal their status to avoid being stigmatized. They believed that the risks of being stigmatized and discriminated against far outweighed the potential gains such as access to social support following their disclosure of HIV status.

Interviewee: People hide because of fear of isolation, people, about 2000 people in Mogadishu live with HIV. But only 600 registered with local services. The rest get their HIV medication through black market. They are hiding, otherwise family and kinship ties get severed. Even the 600 registered, only myself and two others have family support.

The above extract, provided by participants with professional involvement in HIV services in Mogadishu, suggests that many people living with HIV in the city were concealing their disease. Although they accessed treatment, and possibly aspired to a good prognosis, stigma is forcing them underground. HIV stigma is even preventing people from accessing treatment and care, instead opting for non-conventional sources of medication. This has a potential impact on the quality of treatment and care for those living with diagnosed HIV in Mogadishu, because the so-called black market sources may not meet the standard practice requirements. 

### 3.5. Sources of Support 

Although our participants reported various challenges in their daily lives with HIV, as discussed above, they are some sources of support that they relied on. The support they received ranged from material support, such as food and money, to emotional support. We found that there were two main sources of support; close family members and NGOs. The NGOs provided money for food, rent, or clothing. They also provided peer support services where the participants could meet and share their experiences of living with HIV. The close family members commonly cited by the participants were mainly parents, particularly mothers, although one female participant said her main source of financial support and moral support was her father. Some of the participants received material support from their relatives in the diaspora.

Interviewee: For me, my brothers, my aunt´s children in the UK send me money to survive. My other relatives don’t have money for me, but they send me greetings, checking on how am doing, visit me, because they are also poor.

As illustrated above, most of the relatives based in Somali were said to be economically poor and could not offer more than emotional support, which the participants appreciated all the same. However, the participants could not reveal their serostatus to some of their siblings because of the stigma, as outlined above. When some of the participants’ siblings learnt of their HIV status, relationships became strained and the support was withdrawn.

*Interviewee: My mother advised me not to tell my siblings or anyone else. She was afraid I will be stigmatized … the problem I faced was that the few monies I was getting from my siblings were cut off. The 50 to 100 US dollars. But to them it is their ignorance. But I thank God my mother gives me everything.*

Interestingly, some of the participants had not disclosed their HIV status to their siblings, hence the support they received was not because they were living with HIV but because of the socioeconomic challenges the siblings believed they were exposed to in Mogadishu. As illustrated above, there was a risk of losing that support when their HIV positive status was exposed. The participant quoted above for example had become an HIV awareness activist and was open about his condition as part of his efforts to confront HIV stigma. He said he had been interviewed on national TV about living with HIV, and his siblings learnt that he had HIV through the TV broadcast. The result was loss of the support he received from his siblings. Luckily for him, he continued to draw support from his mother. 

### 3.6. Resilience

We found numerous examples of resilience, used here to mean self-coping strategies, that the participants used to manage their daily life with HIV. Despite their daily challenges, the respondents had a sense of a ‘can do attitude’ and were ready to live a complete life as much as their efforts could enable them to, as illustrated below.

Interviewee: My blood is good, my skin looks better, it is shiny and feeling better. I go and wash clothes for people, make about 100 to 120 thousand shillings per day.

The participant quoted above observed that she was in good health and said that she did the best she could to earn money to support herself and her children. This is an example of active coping in the face of adversity which exemplifies the resilience of these participants. They had also formed their own NGO, through which they championed their needs and sought collective support for all of them. Initially, the participants said that the NGO was headed by HIV negative individuals who embezzled funds. They said they revolted against the corrupt HIV negative officials and took control over the NGO in order to represent themselves. The feel-good factor highlighted above, an illustration of resilience, was a common expression among many participants. They did not allow themselves to feel downtrodden because they had HIV, as further illustrated below.

Interviewee: You see how handsome I look today? If I am out there on the streets, nobody can tell what I have. No one looks better than me. Am 100% sure of myself.

The participants were also full of optimism about their prognosis. None of them expressed concerns about their prognosis. Instead, they sought means of securing financial support so that they could live a comfortable life. In their view, it was not a question of whether they were going to live longer but rather how to live a better quality of life. As outlined above, the participants relied on various sources of social and material support to enable them to survive with HIV. They also actively sought further sources of support from the individuals and organizations they interacted with. Although the participants did not actually have access to numerous sources of support, they remained determined to live long and looked forward to the next opportunity that could make their lives with HIV better. 

## 4. Discussion

The participants in this study believed that they acquired HIV through their spouses or through health care-related contamination; they felt that it was not in their means to avoid getting HIV. Getting HIV caused a breakdown in social networks and there was family rejection and depletion of resources. This study reveals the constant struggle experienced by people living with HIV, as previously highlighted with limited resources, little support, and stigma, especially in places of instability [13,14]. 

There was widespread stigma that manifested in the participants being chased away from home. Despite the stigma from their family members, these people received even stronger stigmatization from their communities, a scenario well-reported in previous studies [15]. They could not easily rent houses or had to pay higher rental fees. Loss of work or the sheer snub of their merchandise, for those involved in small-scale trading, by those who knew of their HIV status led to selective disclosure to avoid stigma [16]. Their story of stigma is a consistent with that told by HIV positive women living in the Kibera slums of Kenya [17]. These realities cut across a wider audience when it comes to selling their goods and merchandise. This is why many are reported not to seek care, despite the readily available drugs in health care service centers [18,19]. They are often faced with difficult issues in disclosing their HIV status to their peers and families due to the widespread stigma [20,21,22,23]. They were very conscious about the people with whom they could discuss their condition [24]. From these accounts, it is clear that the social stigma, which was a serious reality when HIV had just been detected, still exists in Mogadishu today [25].

Unlike some earlier studies, there was no stigma reported in accessing medication and health care services, and all participants had access to drugs from NGOs [26]. They did not report any stigma from health care personnel. They adhered to the treatment and were aware of the consequences of failure in adherence to their HIV treatment. Participants reported proper adherence despite the challenges, as reported in other studies [27]. They were also informed that HIV treatment was not a cure, but rather worked to minimize the effects of the disease. They were aware of the nutritional requirements, but could not afford the necessary balanced diet that was meant to enhance the efficacy of their treatment. Most of them sought support from other HIV positive individuals and NGOs. 

In the aftermath of being diagnosed with HIV, the devastating effects and self-pity among these people was not observed, as reported in earlier studies [28]. Even when the treatment was not available in the beginning, they travelled to farther places for their medication. Their resilience and motivation to live a better life compelled them to look for odd jobs to support themselves. They started their own NGO as a coping strategy and were very optimistic about their prognosis. 

The participants lived with each other and shared accommodation as a way to cope with the social pressure, including internal relocations to places they believed were safer [29] from perceived or experienced stigma. The formulation of support groups has been cited to be an effective way of coping with the effects of social stigma [30,31]. A coping and enabling social environment is key in dealing with the hardships that come with HIV infection [32]. The results of this study suggest that, in the event that the stigma reduces and there is an increase in social engagement, the people will cope favorably with their conditions as earlier highlighted [33]. It is worth noting that there is no national budget to cover this program, and the hospital implements HIV programs including drug distribution with assistance from NGOs.

### Methodological Consideration

The paper had a rather small sample which made it very hard to make strong conclusions about the realities of living with HIV in Mogadishu. It would have been ideal to interview both patients doing well on the treatment and those doing badly on the treatment. This would have given deeper insights into the treatment dynamics, but this was not possible.

## 5. Conclusions 

Despite of the instability caused by years of conflict, our findings suggest high level of awareness about HIV and the realities of living with the disease among those living with diagnosed HIV in Mogadishu. This study demonstrated the existence of accessible HIV services that can be accessed by those resilient enough to seek care. Just as in other settings, HIV stigma is big challenge in Somalia, and people living with HIV have to devise various coping strategies. The findings also mirror the global optimism about better prognoses for people living with diagnosed HIV who are on successful treatment, as illustrated by the participants’ upbeat outlook on their future despite their immediate concerns. This study shows that there is need for further research in this context of conflict.
